# Adverse Drug Reactions Caused by Antimicrobials Treatment for Ventilator-Associated Pneumonia

**DOI:** 10.3389/fphar.2022.921307

**Published:** 2022-05-31

**Authors:** Shan Shen, Ning Hou

**Affiliations:** Department of Pharmacy, Shandong Provincial Hospital Affiliated to Shandong First Medical University, Jinan, China

**Keywords:** ventilator-associated pneumonia, antimicrobials, adverse drug reactions, ICU therapy, medication use, drug interaction

## Introduction

Ventilator-associated pneumonia (VAP) develops in intensive care units (ICU) patients who have been mechanically ventilated for at least 48 h, and antimicrobials are an important treatment in clinical management. About 50% of the antimicrobials in ICUs are used for the treatment of VAP, of which the use rate of unnecessary, inappropriate or suboptimal antimicrobials accounts for 30%–60% (Luyt et al., 2014), so the antimicrobial treatment of VAP patients is still a major clinical difficulty. For patients with VAP, inadequate treatment and delay in administering of effective antibacterial drugs are both associated with increased mortality (Muscedere et al., 2012; Swanson and Wells, 2013), and long-term use of broad-spectrum antimicrobials can increase the incidence of adverse drug reactions (ADR) (Fagon et al., 2000) and antimicrobial resistance. Therefore, enhancing management of antimicrobials will be beneficial to reduce the occurrence of ADR, improve the effectiveness of antibacterial drugs treatment, and curb the spreading of drug resistance. This article mainly introduces the cases of ADR caused by antimicrobials treatment of VAP, in order to provide a reference for the safe, effective and rational use of antibacterial drugs in clinical practice.

## Clinical Characteristics of VAP and Selection of Antimicrobials

There are many pathogenic species of VAP, and the main pathogens include Gram-positive and Gram-negative bacteria: *Staphylococcus aureus* (including methicillin-resistant *Staphylococcus aureus*, MRSA), *Acinetobacter baumannii*, *Klebsiella pneumoniae*, *Pseudomonas aeruginosa* (Huang et al., 2018; Luyt et al., 2018). The antimicrobial treatment with VAP is generally divided into two steps: one For patients with an early diagnosis of VAP, antimicrobials should be used empirically by most clinicians. Generally, it is based on the local distribution of pathogens, risk factors for MDR pathogen infection, etc.; two Once the patient’s microbiological examination results are obtained, a corresponding treatment plan should be formulated according to the results of the susceptibilities, and the treatment should be adjusted to narrow-spectrum antimicrobials on time (Luyt et al., 2014).

At present, there are many kinds of antimicrobials for VAP. Ceftazidime, cefepime, piperacillin/tazobactam, levofloxacin can be used for patients without risk factors of multidrug-resistant (MDR) pathogens. New β-lactam antimicrobials (ceftazidime-avibactam) and carbapenems (imipenem/meropenem) are available for the empirical treatment of MDR/extensive drug resistance (XDR) pathogens of VAP. Vancomycin or linezolid should be added if MRSA infection is suspected or present. In conclusion, if there is no risk of MDR infection, the antimicrobials which recommended are relatively narrow-spectrum. If there is a risk of MDR infection, broad-spectrum antimicrobials with anti-pseudomonas effect are recommended, and antimicrobials against MRSA should be added when necessary.

The duration of antimicrobial treatment has always been the focus of clinical attention. The evidence that short-term antimicrobial therapy reduces drug resistance, but the recurrence of drug withdrawal is rare, the advantages of short-term antimicrobial therapy outweigh the disadvantages. Short course of treatment (7 days) for VAP patients rather than 8–15 days is recommended by American guidelines (Kalil et al., 2016). AMMI guidelines indicate that more prolonged periods of time (14 days) was proposed to be used on MDR infection or *Pseudomonas aeruginosa* infection (Rotstein et al., 2008).

## Cases of ADR Caused by VAP Antimicrobials Treatment

VAP is one of the most common ICU-acquired infections and has a high risk of death. Most patients have complicated conditions. Antimicrobial treatment is the cornerstone of VAP treatment, especially for MDR and XDR infections. Articles published within the last 10 years to 1 April 2022 were searched through Pubmed databases. We used ‘Ventilator-associated pneumonia’ as the search terms. The search was restricted to case reports. The database search produced 178 articles. After browsing the abstract of the articles, four were reviewed for the final inclusion according to the theme of the article.

### Case 1 Vancomycin-Induced Thrombocytopenia

A 52-year-old man was admitted to ICU with hypertension and secondary pulmonary edema, requiring endotracheal intubation due to acute hypoxemic respiratory failure, and acute kidney injury. He developed ventilator-associated pneumonia during treatment. But no related pathogens were cultured, so vancomycin was given to empirically treat VAP. Three days later, platelets dropped sharply from 172×10^9^/L to 3×10^9^/L. A presumptive diagnosis of vancomycin induced thrombocytopenia (VIT) was made. All medications were discontinued and methylprednisolone (500 mg/day) and intravenous immune globulin were prescribed. The patient’s platelet count returned to normal after 18 days.Vancomycin-dependent antiplatelet antibodies was identified in patient serum by flow cytometry. But VIT was not completely understood. The antibody bind platelets only in the presence of vancomycin had been described *in vitro* experiments. So the vancomycin-induced thrombocytopenia increased the risk of bleeding. In addition, it took at least 6 days to develop VIT after initial exposure to vancomycin, and an average of 8 days to reach platelet nadir, but the interval between re-exposure to vancomycin was significantly shorter. Since medication of vancomycin had been stopped, 18 days later, the patient’s platelets returned to normal, this is probably caused by the reduction of vancomycin clearance due to acute renal injury. It is suggested that the slow speed of drug clearance should be considered during medication for patients with renal function injury, which avoid serious consequences caused by drug accumulation (Abdalhadi et al., 2020).

### Case 2 Piperacillin/Tazobactam-Induced Platelet Dysfunction

A 73-year-old male patient was in a coma from an out-of-hospital cardiac arrest. After resuscitation, he was admitted to the cardiac intensive care unit (CCU) with mechanical ventilation. On hospital day 5 (HD5), he was diagnosed with *Pseudomonas aeruginosa* ventilator-associated pneumonia with sepsis. The glomerular filtration rate (GFR) was 29 mL/min/1.73 m^2^. Piperacillin/tazobactam (TZP) was given 3×4.5 g daily. On HD 8, the patient developed bleeding symptoms. On HD 14, due to further deterioration of renal function, TZP was adjusted to 3×2.25 g daily. TZP was stopped because it might be the cause of platelet dysfunction. After blood transfusion treatment, the condition was relieved. Although platelet counts and coagulation tests were normal during treatment, platelet function tests showed severely impaired ADP-dependent platelet aggregation. The reason is most likely the toxicity of TZP, which is exacerbated by drug accumulation during the progression of renal failure. It suggests that regular monitoring of renal function is very important (Skoric et al., 2020).

### Case 3 Ciprofloxacin-Induced Rhabdomyolysis

A 64-year-old male patient was intubated and given mechanical ventilation for subacute non-ST elevation myocardial infarction (NSTEMI) and pulmonary edema. Empirical antimicrobial treatment was started due to the increase of C-reactive protein (CRP). A travel history in a country at risk of carbapenem-resistant *Enterobacteriaceae* (CRE), and the drug susceptibility test showed low sensitivity to carbapenems and sensitivity to ciprofloxacin, so ciprofloxacin 400 mg q8h was given. Creatine kinase (CK) levels increased to 4,981 U/l Ciprofloxacin was suspected as causative for rhabdomyolysis and it was discontinued immediately. The patient’s creatine kinase levels began to increase significantly after increasing the dose of ciprofloxacin, possibly due to fluoroquinolone-induced rhabdomyolysis. This report highlights the global spread of carbapenem-resistant Enterobacteriaceae. Clinicians need to be aware that some common antimicrobials cause ADR. For example, these drugs may lead to rhabdomyolysis (Grisold et al., 2013).

### Case 4 Cefepime Associated With Phenytoin-Induced Stevens-Johnson Syndrome

A 49-year-old man with sepsis and renal failure suffered from a generalized status epilepticus during treatment of VAP with cefepime, then phenytoin was added to treatment. Cefepime was discontinued due to its epileptogenic potential, after 24 h under treatment with phenytoin, the patient developed macular rash on the trunk and lower extremities. This symptom is consistent with Stevens-Johnson syndrome, there was drug interaction probably between cefepime and phenytoin. The cause of drug interaction might be the immune response triggered by a reaction between water nucleophilic groups of phenytoin and carbonyl group of the cefepime, forming cephalosporic acid which leads to cephalosporin derived proteins. At the same time, due to the renal damage of the patient, the complete elimination time of cefepime from the body is prolonged, which induces the coexistence of the two drugs in the body, and causes pharmacological interaction. In addition, cephalosporins themselves may also contribute to these clinical manifestations. It can be seen that doctors and pharmacists need pay attention to the interaction between drugs during treatment (Marco-Del Río et al., 2017).

Naranjo’s algorithm or Drug Interaction Probability Scale (DIPS) was used to assess the causal relationship between the suspected drug and drug reaction. According to this system, the ADR in the four reports could be categorized as probable (ADR probability score 6–8). Among them, 2 (case 3 and case 4)has causality evaluation records in the original literature.

At present, most antimicrobials need to be excreted through the kidney, and some of them have nephrotoxicity. In the above cases, the three patients (case 1, case 2 and case4) were accompanied by renal function injury in the ADR, which may lead to accumulation of drugs and prolong its’ time to eliminate from the body, increase toxic and side effects of drugs. These may be would cause increase the possibility of ADR. Based on the complexity of VAP and the underlying diseases of the patients, careful selection of antibacterial is required for special populations. With the increased resistance of pathogenic microorganisms to antimicrobial drugs, some commonly antimicrobials have been reintroduced. It is that seems to have good effective on VAP. But the ADR of common antimicrobials may be ignored, thus increasing the occurrence of adverse reactions (case 3). In addition to the inherent toxic and side effects of drugs, the treatment is frequently accompanied by a variety of drugs in the case of complex conditions, which drug interactions may be occur inevitably. In case 4, phenytoin is a drug that must be closely monitored. It is a great inductor of hepatic microsomal enzymes and can reduce the plasmatic levels of other drugs. Therefore, the possibility of pharmacokinetic interaction need to be considered when combined with other drugs. VAP with MDR or XDR, the combined use of multiple antibacterial drugs can be considered. But the combined use of drugs may change the pharmacokinetic characteristics, especially when multiple drugs are used together and the patient’s liver and kidney functions are damaged, which may increase the occurrence of ADR. In this case, we should pay close attention to the changes in the corresponding indicators and monitor the medication timely. For patients with other complicated conditions, drug susceptibility testing, evidence-based medicine, and pharmacokinetic theory can be combined with clinical diagnosis to judge the risk and benefit of the patient. In addition, the study found that antimicrobial peptides provide a new alternative, which is a potential therapeutic approach to overcome antibiotic resistance, improve killing efficacy and reduce side effects (Zhang et al., 2021). At the same time, we need to be aware the relevant mechanisms of antimicrobial resistance. According to the Shen’s report, model conjugative plasmids can help us understand the spread of antimicrobial resistance, which will be beneficial to ensure the therapeutic effect and safety of antimicrobial in clinical application (Shen et al., 2022). In conclusion,optimal antimicrobial therapy for VAP treatment remains inidentified. Clinicians and clinical pharmacists should take new strategy approach to old drugs and make effective utilization of existing resources to reduce the possible antimicrobial resistance.

This paper summarizes and analyzes the adverse reactions related to the clinical antimicrobial treatment of VAP in the retrieved cases, reminds doctors and clinical pharmacists to pay attention to adverse antibacterials reaction and monitoring the patient during medication use, therapeutic regimen of VAP need to be supervised closely in clinical practice.
